# Role of Dectin-1 in the innate immune response of rat corneal epithelial cells to *Aspergillus fumigatus*

**DOI:** 10.1186/s12886-015-0112-1

**Published:** 2015-10-01

**Authors:** Qiang Xu, Guiqiu Zhao, Jing Lin, Qian Wang, Liting Hu, Zhao Jiang

**Affiliations:** Affiliated Hospital of Qingdao University, 16th Jiangsu Road, Qingdao, Shandong Province China

**Keywords:** Dectin-1, Innate immune, Corneal epithelium, Rat fungal keratitis, Laminarin

## Abstract

**Background:**

To observe Dectin-1 expression in fungal keratitis on rat models and to determine the role of Dectin-1 in innate immune response to *Aspergillus fumigatus*.

**Methods:**

Wistar rats were randomly divided into control, fungal keratitis and pretreatment (pretreated with Laminarin) groups. Samples were used for conducting immunohistochemical staining and real-time PCR to observe expression of cytokines like CCL2, CCL3, CXCL1, CXCL2, IL-1β, TNF-α, IL-6, IL-10.

**Results:**

After fungal stimulations, all 7 inflammatory factors, except IL-10, increased with different levels. After 4 h of fungal stimulations, IL-1β, IL-6, CCL2, CXCL1 and CXCL2 of pretreatment groups were significantly (*p* < 0.05) lower than fungal groups, while the other 3 cytokines had no significant changes. After 8 h of fungal stimulations, IL-6 and CXCL1 of pretreatment groups were still significantly (*p* < 0.05) lower than fungal groups.

**Discussion:**

With progress of fungus stimulation, expression of IL-1β,CXCL1 ,CXCL2,MCP-1 gradually increased, whilepretreated with Laminarin to block Dectin-1, these expression decreased, indicating that Dectin-1 maypromote immune reaction through them. IL-10 decreased in fungal group because of itsimmunosuppressive effect at 4h, and it began to increase at 8h to suppress Th1 inflammation response inorder to avoid excessive tissue damage.

**Conclusion:**

Dectin-1 in early period of innate immune responses in rat fungal keratitis might work through IL-1β, IL-6, CCL2, CXCL1, CXCL2 to recruit neutrophils and macrophages to participate anti-fungal immunity.

## Background

Fungal keratitis (FK) is a serious eye disease which leads to blindness in some areas in China, especially for young adults. Therefore, it is very important to determine the pathogenesis of this disease and to find effective therapeutic targets. As the first line of defense in the immune response, innate immunity plays an important role in human defense of fungal infection. Pattern recognition receptors (PRRs) are key factors of innate immune response system, which could recognize pathogen-associated molecular patterns (PAMPs) and start a quick innate immune response, and then trigger persistent, specific adaptive immune response. Dectin-1, one of the β-glucan receptor expressed in dendritic cells and macrophages plays a vital role in anti-fungal infection by recognizing fungi, recruiting immune cells, releasing inflammatory cytokines and starting an adaptive immune response.

Studies have shown that non-immune cells such as corneal epithelial cells and stromal cells may participate in innate immune responses through their own pattern recognition receptors. As the first line of defense, ocular corneal epithelium is not only a mechanical barrier to protect from the invasion of fungi, but also can identify the fungus through pattern recognition receptors [1]. Recent studies show that Dectin-1 plays an important role in the defense of the fungal infection [2]. Dectin-1 in the cornea can be unregulated after *Fusarium* and *Aspergillus* infection, while the Dectin-1 knockout rat is susceptible to *Candida albicans* and *Aspergillus*. When suffering from pulmonary *Aspergillus fumigatus* infection, Dectin-1 knockout rat had less lung inflammation response and more fungal load. Therefore, Dectin-1 expressed on corneal dendritic cells and macrophages play an important role in innate immune response by the early identification of *Aspergillus fumigatus*, recruitment of immune cells and release of inflammatory factors.

In this study, we pretreated rat corneal epithelium with Dectin-1 inhibitor laminarin (Laminarin), and then created fungal keratitis on rat models. The expressions of eight inflammatory factor (CCL2, CCL3, CXCL1, CXCL2, IL-1β, TNF-α, IL-6, IL-10) were monitored by immunohistochemistry and Real-time RT-PCR method in order to determine the role that Dectin-1 plays in early recruitment of inflammatory cells and to find its relationship with subsequent adaptive immunity response in fungal infections of the cornea.

## Methods

### Source of rat and fungal strains

All Wistar rats were approved by Qingdao medicine inspecting institute, Shandong province. They were healthy, ophthalmic diseases free, and treated in accordance with the guidelines provided in Scientific and Technological Commission of China for the Use of Animals for lab Research. Standard *Aspergillus fumigatus* strains (NO 3.0772) used in this study were purchased from China General Microbiological Culture Collection Center (CGMCC), and cultured according to standard procedure.

### Establishment of a rat model of *Aspergillus* keratitis

Rat were randomly divided into control group (*n* = 6) and fungal keratitis group (*n* = 48). The fungal keratitis groups were divided into pretreatment group (*n* = 24) and fungi positive control group (*n* = 24). Pretreatment groups were pretreated with Laminarin eye dropping solution (20 mg/mL) for 2 h, and fungal keratitis models were created at the same time with fungal keratitis group. The model was established and evaluated according to the criteria reported by Li et al. [3]. Corneal epithelium was collected at 4, 8, 16 and 24 h after the experimental model established for real-time PCR analysis. Eyeballs were collected at 24 h for immunohistochemistry.

### Real-time PCR

Trizol Reagent was purchased from Invitrigen Prime Script RT reagent Kit With gDNA Eraser (Perfect Real Time) was purchased from TaKaRa. The house keeping gene GAPDH, primers and probes were purchased from TaKaRa. The sequences of the primers used were listed in Table 1.

**Table 1:** Sequence of GAPDH, CCL2, CCL3, CXCL1, CXCL2, IL-1β, TNF-α, IL-6 and IL-10

	Sequence sequence (5' → 3')		Gene bank
GAPDH	Sense	CCCCCAATGTATCCGTTGTG	
	Antisense	GTAGCCCAGGATGCCCTTTAGT	NM_017008
	Probe	TCTGACATGCCGCCTGGAGAAACC	
CCL2	Sense	ACCCATAAATCTGAAGCTA	
	Antisense	GCATCACATTCCAAATCA	NM_031530.1
	Probe	TCCACAACCACCTCAAGCACT	
CCL3	Sense	GTCACATTTGTGTTTGTAG	
	Antisense	CCTAGAATAATTGTCACCAA	NM_013025.2
	Probe	AAAGACCTCAGGGCACATTCC	
CXCL1	Sense	GGCTTCTGACAACACTAG	
	Antisense	ACGAGATATTTAACGCCTAC	NM_030845.1
	Probe	CTGCACAATTGGAATTGAACGACCA	
CXCL2	Sense	GTGCCTAGATGTTGTTAC	
	Antisense	CCTTCCAACTACATAAGTAA	NM_053647.1
	Probe	ATGCTGACTGAACACATTGAACATT	
IL-1β	Sense	CTTCGAGATGAACAACAA	
	Antisense	CATGGAGAATACCACTTG	NM_031512.2
	Probe	ATGCCTCGTGCTGTCTGACC	
TNF-α	Sense	CTGTCTACTGAACTTCGG	
	Antisense	CATGGAACTGATGAGAGG	HQ201305.1
	Probe	TCCCAACAAGGAGGAGAAGTTCC	
IL-6	Sense	TCAGGAACAGCTATGAAG	
	Antisense	AGTGGTATATACTGGTCTG	M26744.1
	Probe	CTTCCAGCCAGTTGCCTTCTTG	
IL-10	Sense	GATCCAGAGATCTTAGCTA	
	Antisense	CTGAGGTATCAGAGGTAA	L02926.1
	Probe	AACCTCGTTTGTACCTCTCTCCAA	

To minimize the experimental error, total RNA was synchronously extracted from the corneal epithelium samples obtained at different observation points using Trizol reagent according to the manufacturer's protocol. Measurements of RNA concentration, reverse transcription and real-time quantitative PCR reactions were conducted in sequence.

### Immunocytochemistry

Rabbit anti-rats CCL2, CCL3, CXCL1, CXCL2, IL-1β, TNF-α, IL-6, and IL-10 multi-clonal antibody, Histostain PLUS kit and DAB kit were purchased from Beijing Biosynthesis Biotechnology Co., Ltd.

Corneal paraffin sections, the thickness of 2 μm, were conventional dewaxed to water. S-P method was applied to stain every cytokine in corneal tissue. PBS buffer was used as negative control. Corneal tissue appeared brown particles for positive criteria. Field of vision was randomly selected and saved under 200 times zoom, the mean optical density of every cytokine staining was analyzed with VIDAS-21 (Japan SANYO) the computer color image analysis system.

### Statistical analysis

All data were presented as mean ± SD (*n* = 6). The data were analyzed with SPSS17.0 statistical package. Single factor analysis of variance was used to collectively comparison. The LSD tests were used to pairwise comparisons. The differences between groups were analyzed by T test. *P* <0.05 was considered to be statistically significant.

## Results

### Immunocytochemistry

**Fig. 1:**
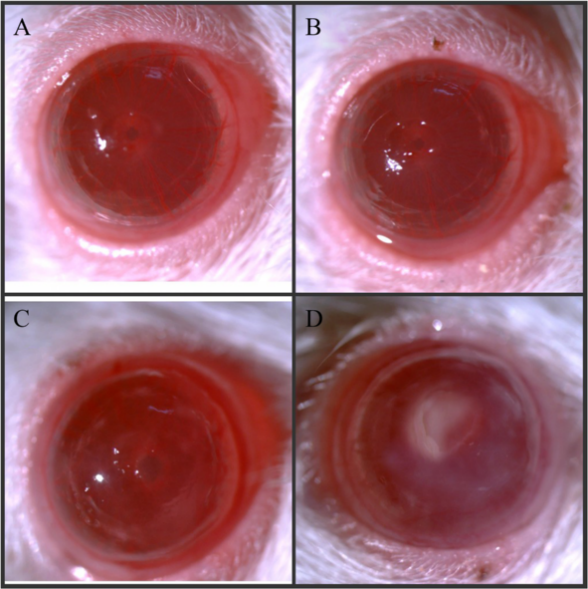
Morphology of fungal infections models. **a** 4 h after fungal infection **b** 8 h after fungal infection **c** 16 h after fungal infection **d** 24 h after fungal infection

**Fig. 2:**
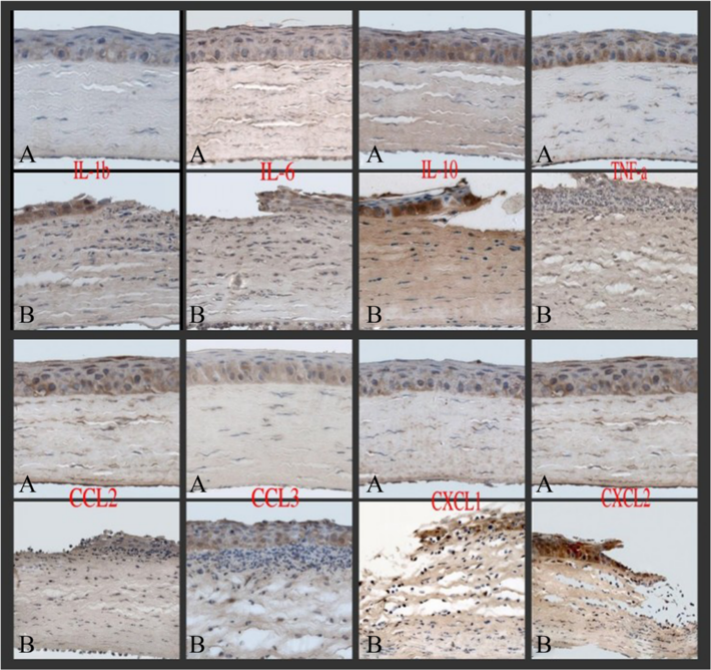
Immunocytochemistry results immunohistochemical staining × 400. **a** normal rat group **b** fungal group. The eight inflammatory factors were mainly expressed in cytoplasm in the corneal epithelium and shallow stroma. The fungal groups and pretreatment groups showed clear pictures by comparing with normal rat groups, while there were no obvious difference between fungal groups and pretreatment groups

The changes of corneas on rat models after fungal stimulation in different time points were observed as showed in Fig. 1. Positive results of immunohistochemical staining can be seen in the cell. The greater inflammatory factors had deeper color in tissues. Results demonstrated that eight inflammatory factors were mainly expressed in cytoplasm in the corneal epithelium and shallow stroma. The fungal groups and pretreatment groups showed clear pictures by comparing with normal rat groups, while there were no obvious difference between fungal groups and pretreatment groups (Fig. 2).

### Real-time RT-PCR

**Fig. 3:**
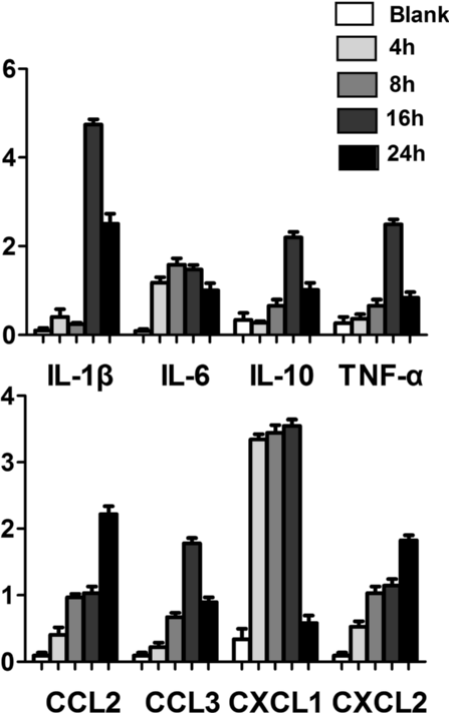
Real-time RT-PCR results. After fungal stimulations, all 7 inflammatory factors, except IL-10, increased with different degrees, during which, IL-1β, TNF-α, CCL3 peaked at 16 h and decreased at 24 h, CXCL1 peaked at 4 h, IL-6 peaked at 8 h, CCL2 and CXCL2 continued rising. IL-10 decreased at 4 h, increased from 8 h, peaked at 16 h, and reduced from 24 h

**Fig. 4:**
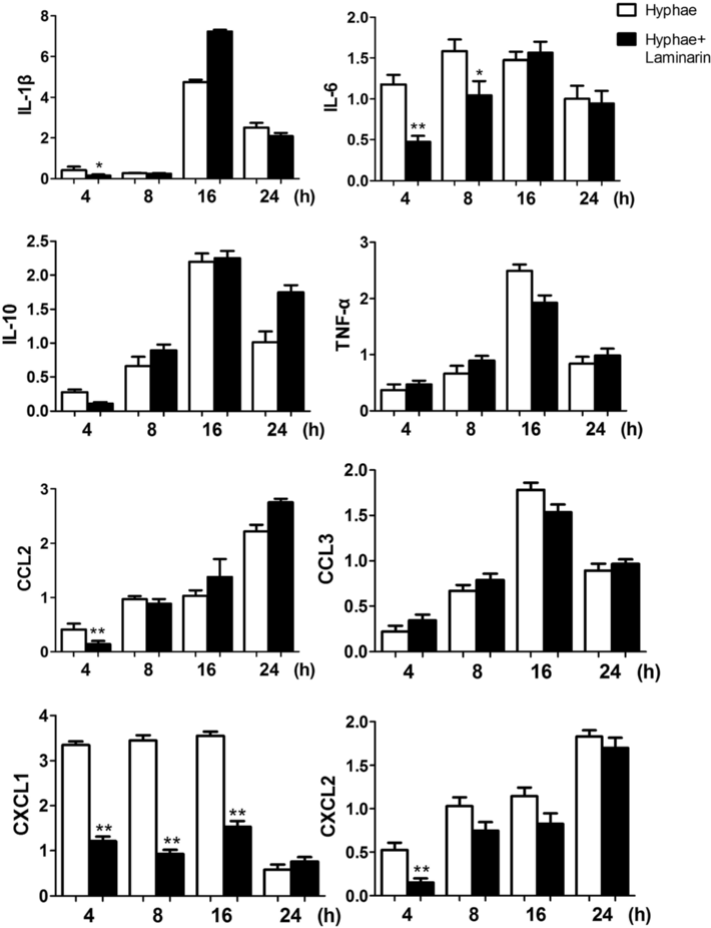
Real-time RT-PCR results. After 4 h of fungal stimulations, IL-1β, IL-6, CCL2, CXCL1 and CXCL2 of pretreatment groups were lower than fungus groups and was statistically significant (*p* < 0.05), while the other 3 factors with no statistically significant. After 8 h of fungal stimulations, IL-6 and CXCL1 of pretreatment groups were still lower than fungus groups and was statistically significant (*p* < 0.05)

As shown in Figs. 3 and 4, expression of eight inflammatory factors mRNA was detected in every blank (normal rat) group. After 4 h of fungus stimulation, expression of IL-1β, IL-6, CCL2, CXCL1, CXCL2 increased and had significant difference (*p* < 0.05). After 8 h of fungus stimulation, expression of TNF-α and CCL3 increased more and had significant difference (*p* < 0.05). Expression of IL-10 was lower in 4 h fungus groups than control groups, and at 8 h fungus groups it increased and was statistically significant compared with control groups (*p* < 0.05). IL-1β, TNF-α and CCL3 reached maximum at 16 h, and then decreased from 24 h. CXCL1 increased from 4 h to 16 h, and then decreased. IL-6 reached peak at 8 h, then gradually decreased, while expression of CCL2 and CXCL2 continued to increase for all the time.

## Discussion

Laminarin, 1,3-β-glucan extracted from a Laminoriadigitata, can specifically bind Dectin-1. It is considered as Dectin-1 specific blocker. The study indicated that Laminarin connected with ovalbumin OVA could be specially recognized by Dectin-1 receptor expressed on dendritic cells and macrophage. CD4^+^ T cell responses could be induced through cross-presentation, leading to secretion of inflammatory factors by dendritic cells, which can verify that Laminarin could act as Dectin-1 specific blocker in the animal models [4].

Dectin-1 can recognize particulate and soluble β-glucans from fungi, bacteria, and plants, and is the primary receptor for these carbohydrates on leukocytes, through which it can induce the innate immune reaction and eradicate pathogens. Dectin-1 is expressed predominantly by myeloid cells in many tissues, with highest levels of expression on inflammatory cells and cells at portals of pathogen entry, such as alveolar macrophages [5]. In addition to macrophages, Dectin-1 is expressed on monocytes, neutrophils, and most subsets of dendritic cells as well as on subpopulations of T cells. Ocular Dectin-1 is mainly expressed in the corneal epithelial cells, macrophages and dendritic cells [6].

It was found that the susceptibility to *Candida alricans* in Dectin-1^−/−^ rat was declined with low level of inflammatory reaction and decreased fungal exterminating ability [7]. Many studies showed that Dectin-1 is able to induce ligand uptake by endocytosis and phagocytosis, the respiratory burst, the production of arachidonic acid metabolites, and the induction of numerous cytokines and chemokines. Spleen Tyrosine Kinase (Syk) is a central kinase and mediates most of the functions of Dectin-1 and this signaling pathways plays a critical role [8]. Signaling downstream from Syk involves the novel adaptor CARD9, and activation of mitogen-activated protein (MAP) kinases, nuclear factor of activated T cells (NFAT) and nuclear factor kappa B (NF-κB) [9–11] which finally results in expression of downstream factors like, including TNF, CXCL2, IL-23, IL-6, IL-10, IL-2, and IL-12 [12].

Studies have proved that Dectin-1 gene and protein can express in normal human corneal epithelium, and after *Aspergillus fumigatus* stimulation, synthesis of Dectin-1 was increased, suggesting that Dectin-1 could be activated in fungal keratitis. Leal et al. [2] has established a Dectin-1 deficient rat fungal keratitis models. They found that cell infiltration and fungi scavenging were significantly reduced compared with the control group, with decreased production of IL-1β and CXCL1. Therefore, it was thought that dectin-l had an important role in *Aspergillus* fungal keratitis, cytokine production, neutrophil and monocyte recruitment to the corneal stroma. The fungal killing is dependent on the presence of macrophages and dendritic cells, and on expression of Dectin-1.

Our data showed that IL-1β expression level was very low in the control group, and there was not significant increase in corneal epithelium in fungal groups at 4 h, but its expression was significantly increased after 8 h. We assumed that IL-1β was mainly produced by macrophages and monocytes, while raising these cells took some time after corneal fungal infection. We also found that expression of IL-1β lagged compared with monocyte chemoattractant CXCL1 and CXCL2. Pretreated with Laminarin to block Dectin-1 in corneal epithelium, IL-1β expression decreased significantly at 4 h after fungal stimulation.

The main biological activity of IL-10 is immunosuppressive, which can inhibit activation and effector function of T cell and monocyte-macrophage cells. Our study showed that IL-10 higher expression in blank group, and it decreased in fungal group at 4 h. As a Th2-type cytokine, its expression in normal corneal epithelium could inhibit activation of immune cells to prevent immune response leading to tissue damage, and after fungal infections, innate immunity of the body is firstly activated, when the site of infection requires a lot of inflammatory cell, expression of IL-10 decreases to let its immunosuppressive effect down to help killing the fungus early. With progress of infection, immune response was constantly enhanced and expression of IL-10 began to increase at 8 h to suppress Th1 inflammation response in order to avoid excessive tissue damage. Pretreated with Laminarin could block Dectin-1 in corneal epithelium, thus IL-10 expression did not change after fungal stimulation.

Chemokines, mainly composed of macrophages, immune cells and non-immune cells, are involved in inflammatory process by leukocyte chemotactic and chemokinesis. Our study showed that with fungal stimulating, corneal epithelium expression of MIP-2 gradually increased, and pretreated with Laminarin to block Dectin-1 in corneal epithelium, expression of CXCL1 and CXCL2 decreased in 4 h of fungal stimulation, while CXCL1 was still less in pretreatment group than fungal group after 8 h, and the difference was statistically significant, indicating that Dectin-1 may have neutrophil chemotaxis through both CXCL1 and CXCL2.

Monocyte chemotactic protein MCP-1, also known as CCL2, is major monocyte chemotaxis factor in inflammation. It can recruit monocytes, memory T cells and dendritic cells to the site of injury, infection and inflammation, then initiate factor of inflammatory cytokine network. Our results showed that with progress of fungus stimulation, expression of MCP-1 in corneal epithelium gradually increased, and pretreated with Laminarin to block Dectin-1 in corneal epithelium, expression of MCP-1 decreased at 4 h, which was statistically significant, indicating that Dectin-1 may raise monocyte through MCP-1.

## Conclusions

Our experiment confirmed that Dectin-1 in early period of innate immune in rat fungal keratitis can work through IL-1β, IL-6, CCL2, CXCL1, CXCL2 to recruit neutrophils and macrophages to participate anti-fungal immunity. Cytokine production, neutrophil and monocyte recruitment and killing fungi are dependent on presence of macrophage and dendritic cells and expression of β-glucan receptor Dectin-1. Dectin-1, as specific innate immunity molecule modulators in development of this disease, would become targets for antifungal therapy.
